# Patterns and controls of temperature sensitivity of soil respiration in a meadow steppe of the Songnen Plain, Northeast China

**DOI:** 10.1371/journal.pone.0204053

**Published:** 2018-09-24

**Authors:** Ming Wang, Xiujun Li, Shengzhong Wang, Guodong Wang, Jitao Zhang

**Affiliations:** 1 State Environmental Protection Key Laboratory of Wetland Ecology and Vegetation Restoration, Institute for Peat and Mire Research, Northeast Normal University, Changchun, Jilin, China; 2 Key Laboratory of Wetland Ecology and Environment, Northeast Institute of Geography and Agroecology, Chinese Academy of Sciences, Changchun, Jilin, China; 3 Jilin Provincial Key Laboratory for Wetland Ecological Processes and Environmental Change in the Changbai Mountains, Changchun, Jilin, China; Tennessee State University, UNITED STATES

## Abstract

Quantifying the temporal and spatial patterns of temperature sensitivity (*Q*_10_) of soil respiration (Rs) as well as its controlling factors is critical to reveal the response the soil ecological processes to global warming and improve carbon budget estimations at a regional scale. The seasonal and annual variations in the temperature response of Rs were assessed during the two growing seasons in 2011 and 2012 in four different vegetation sites in a meadow steppe of the Songnen Plain, China. The *Q*_10_ values across all sites exhibited significant seasonal variations with a minimum value (1.81–2.34) occurring during summer and a peak value (3.82–4.54) occurring in either spring or autumn. The mean seasonal *Q*_10_ values showed no significant differences among the four different vegetation types. On the annual scale, however, the *Chloris virgata* site had significantly higher annual *Q*_10_ values (3.67–4.22) than the other three community sites in 2011 and 2012 and over the two years (2.01–3.67), indicating that the response of the Rs to climate warming may vary with vegetation type. The soil temperature and moisture had interactive effects on the variations of *Q*_10_ values. Soil temperature was the dominant factor influencing *Q*_10_ values, while soil moisture was an additional contributor to the variations of *Q*_10_. Due to the significant temporal and spatial variations in soil respiration response to temperature, acclimation of Rs to temperature variation should be taken into account in forecasting future terrestrial carbon cycle and its feedback to global warming.

## Introduction

Soil respiration (Rs) is an important component of the terrestrial ecosystems carbon cycle, accounting for about 25% of the global CO_2_ exchange [1] and 60%-90% of total respiration of global terrestrial ecosystems [2]. A small change in the magnitude of Rs could have a large effect on the concentration of CO_2_ in the atmosphere [3]. By the end of this century, global mean temperature is predicted to increase by at least 0.3°C [4]. One of the greatest challenges in predicting the global climate change is to understand how Rs will change with global warming. Studies have indicated that Rs depended strongly on temperature [4–7]. Therefore, the temperature sensitivity of soil respiration (i.e., *Q*_10_ value), which refers to the proportional change in respiration resulting from a 10°C increase in temperature, is typically an important parameter that has been widely used to express the associated relationship between the soil CO_2_ emission from terrestrial ecosystems and climate change.

Even as a reliable predictor in global carbon cycle simulation, *Q*_10_ was often treated as a constant in many ecosystem models, for instance, a constant of 2 or 2.5 used in CASA, TEM and BIOME-BGC models [8–10]. Many field experiments, however, showed that *Q*_10_ values varied temporally and spatially [5,11,12]. It is well known that besides temperature, many other factors, such as the seasonal or annual variations in soil moisture, plant phenology, plant types, microbial populations, enzyme activity, substrate availability and other seasonally fluctuating conditions and processes, could also affect the annual *Q*_10_ values [12–14]. All these factors are spatially and temporally heterogeneous. Therefore, *Q*_10_ values obtained from field measurements potentially vary depending on where and when sampling occurred. This variability of *Q*_10_ value can be especially large in semi-arid areas, where the distribution of vegetation, water condition and belowground soil properties are markedly patchy [15]. In addition, because of the relatively small organic C pools that the semi-arid areas contain, Rs is one of the ecosystem processes most sensitive to climate change [16]. It has been suggested that a small deviation of the *Q*_10_ value in global carbon cycle models may cause an enormous bias in estimating the magnitude of soil CO_2_ release into atmosphere [6, 17]. As such, without considering the temporal and spatial variations, the application of *Q*_10_ in process-based models to predict future soil CO_2_ efflux in response to global warming could result in significant errors. Therefore, a better understanding of *Q*_10_ values at both spatial and temporal scales as well as controlling factors will not only help to reveal the response of the soil ecological processes to global warming, but also benefit for improving the accuracy of carbon budget estimation at regional scales, especially at the semi-arid areas.

Grasslands in China account for nearly 40% of the total country land area and 78% of them are distributed in arid and semi-arid area [18], which plays an important role in the regional carbon cycle [19]. As one of the most important meadow steppes in China, the vast steppe in the western Songnen Plain is a typical semi-arid grassland of China. However, studies regarding soil respiration from this meadow steppe, especially the *in-situ* field study on soil respiration and its temperature sensitivity are still scarce. Therefore, the objectives of this study were to analyze the temperature response of Rs, and to determine the temporal and spatial variation patterns of *Q*_10_ in a temperate semi-arid meadow steppe ecosystem in China. We measured Rs continuously over a two-year period (2011–2012) at four sites dominated by different plant communities. We also assessed the environmental factors that influenced the *Q*_10_ values.

## Materials and methods

### Site description

**Fig 1:**
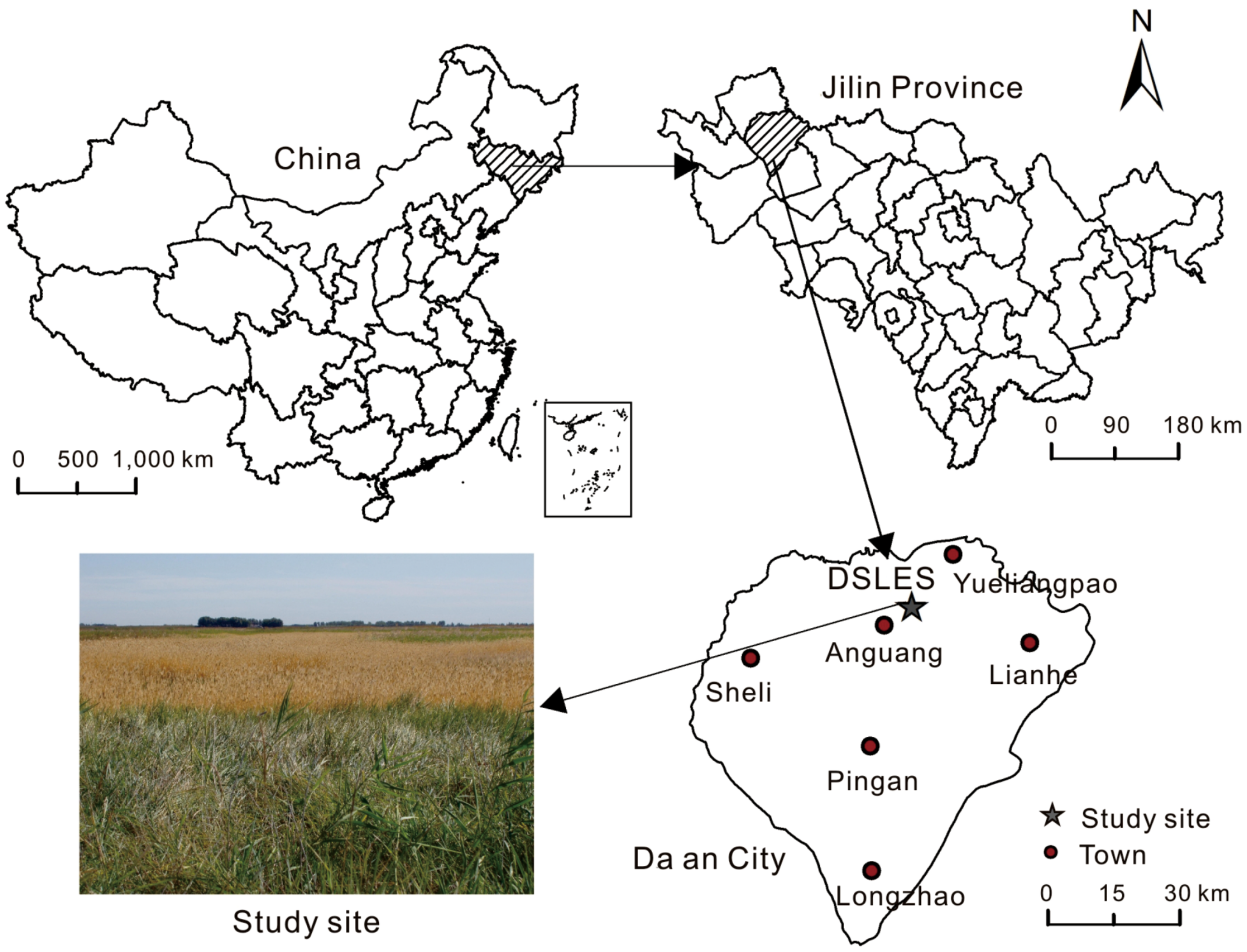
A map of the study site.

**Fig 2:**
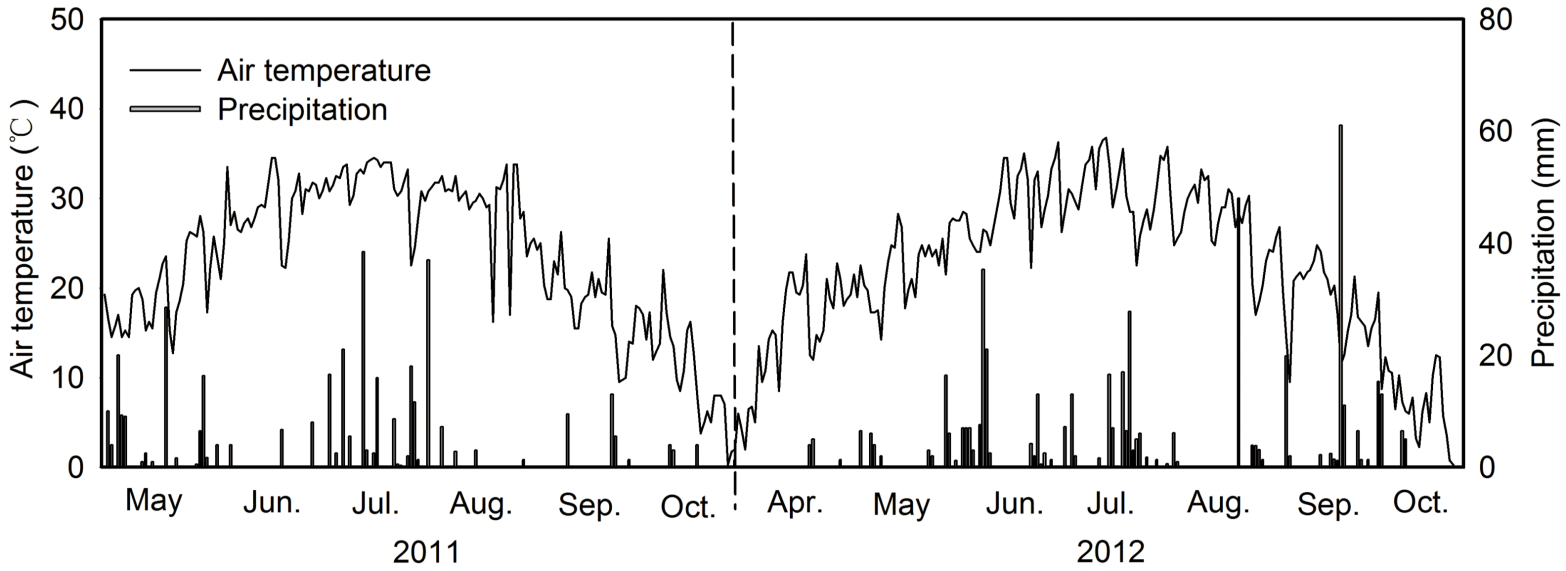
Variation of precipitation (mm) and air temperature (°C) over the experimental period from 2011 to 2012.

This study was conducted at the Da’an Sodic Land Experiment Station of China (DSLES, 45°35′58″-45°36′28″N, 123°50′27″-123°51′31″E), located in the western Songnen Plain (Fig 1). It belongs to a typical temperate meadow steppe ecosystem and is the transitional zone between semi-humid and semi-arid regions. Climate in the region is classified as continental monsoon, with mean annual temperature of 3–5 °C, and mean annual precipitation of 413.7 mm, of which occurred primarily between July and September [20]. In this region, the growing season usually starts in early May and ends in late October, and could be divided into spring (May), summer (June, July, August) and autumn (September, October) for this study. The mean air temperature during the growing season from May to October was similar for 2011 and 2012, at 18.4 °C and 19.5 °C, respectively, but the precipitation was fairly different, with the total precipitation being 370 mm and 498 mm, respectively, in 2011 and 2012 (Fig 2). The soil type in the study site is sodic meadow soil. The vegetation represents naturally regenerated temperate grasses in this region. *Chloris virgata* (CV), *Puccinellia distans* (PD), *Leymus chinensis* (LC) and *Phragmites australis* (PA) are dominant species in the four most common vegetation communities in the study region, representing a sere including primary plant succession and secondary plant succession. For further information regarding plant succession see Wang *et al*. [21]. In this study, we primarily focused on the variation of *Q*_10_ with different vegetation types, without specifically addressing successional transition. The site characteristics and vegetation composition of the study sites in this study are summarized in Table 1.

**Table 1:** Site characteristics and mean soil respiration rate.

Plant community	Site code	Rs (μmol m^-2^ s^-1^)	Ts (°C)	Ws (% v/v)	pH	SOC (g kg^-1^)	TN (g kg^−1^)	Biomass (g m^-2^)
*Chloris virgata*	CV	1.47±0.12	17.9±0.5	22.4±3.2	9.7±0.32	11.9±0.5	0.70±0.04	306.8±17.3
*Puccinellia distans*	PD	1.48±0.17	18.3±0.7	23.9±4.1	9.9±0.02	10.2±2.1	0.61±0.05	190.9±16.7
*Leymus chinensis*	LC	2.38±0.36	17.8±0.5	22.4±3.2	9.4±0.31	15.2±0.1	0.69±0.03	419.3±17.5
*Phragmites australis*	PA	2.76±0.42	16.5±0.4	27.7±3.3	8.4±0.18	18.9±1.4	1.01±0.08	548.8±48.8

Rs, Ts, Ws, pH, SOC, TN are the mean soil respiration rate during the experimental period, the mean soil temperature at 10 cm during the experimental period, the mean soil water content at 0–10 cm during the experimental period, soil pH at 0–20 cm, soil organic carbon at 0–20 cm, and total soil nitrogen at 0–20 cm, respectively. Biomass, means above plant biomass of stand. Values are the means ± SE.

### Field measurements

Each community site had an area no less than 50×50 m^2^. Three 5×5 m^2^ plots were randomly located within each community site, and they were at least 20 m from stand discontinuities or its boundaries. At each plot, three or four PVC collars (10.2 cm inside diameter × 5 cm height) were set randomly and the aboveground vegetation and the litters in the collar were removed 1 day or 2 days before measurements. Soil respiration was measured with a portable CO_2_ infrared gas analyzer (Li-6400) equipped with a Li-6400-09 chamber (Li-Cor Inc, Lincoln, NE, USA). For measurements, all chambers were placed on collars, which had been inserted 3 cm deep into the soil at least 24 h prior to measurement to avoid disturbing the soil with the soil chamber when a measurement was made. The mean Rs value for each collar was the average of three values generated from three continuous measurement cycles at each collar. To achieve a more accurate estimate of Rs, each measurement was repeated and the mean was used in calculations. More information of the monitoring process can be found in Wang *et al*. [21].

Measurements were conducted monthly during the 2011–2012 growing seasons (from May to October), resulting in 12 times in total. On each measurement day, we conducted the 24-h measurement of Rs at an interval of every 2 h, usually beginning at 07:00 am and ending at 07:00 am the next morning. In the winter, the instrument failed to measure the Rs due to very low temperatures. During each measurement period, soil temperature (°C, Ts, 10-cm depth) and soil water content (% w/w, Ws, 0–10 cm depth) were measured near each soil collar. The methods of measuring Ts and Ws were described in the study of Wang *et al*. [21]. Three soil pits with depth to 20 cm were randomly dug in the buffer area of each plot in July 2011 and 2012. A 100 cm^3^ soil column was sampled at 0–10 and 10–20 cm depth, respectively. In the laboratory, soil samples of the two depths were passed through a 2-mm sieve to remove stones and plant fragments, and mixed thoroughly. A weight of 500 g soil sample was taken from the mixed soil sample to measure the concentration of soil organic carbon (SOC) and others soil properties. SOC, soil total N and soil pH in the top 20 cm depth were measured followed the method described by Lu [22].

Live aboveground plant biomass was measured by the harvest method in autumn, 2011 and 2012. At the plot of each site, three 50cm×50cm quadrats were randomly chosen and all plants in the quadrat were clipped down to the soil surface. Live plant parts of the samples were separated manually and then were dried at 70°C in a drying oven to a constant weight. The living and dormant fractions were distinguished by color, texture and shape.

### Data analysis

The *Q*_10_ values used in this study were calculated according to the following equations [23].

$$R_{10}=ae^{bTs}$$ (1)

where Ts is soil temperature, a and b are fitted data-specific parameters. *R*_10_ is the respiration rate at a reference temperature of 10 °C.

The *Q*_10_ value was calculated as:

$$Q_{10}=e^{10b}$$ (2)

To quantify the temporal variability in *Q*_10_ values, the coefficient of variation (C_V_) was calculated as:

$$Cv=\frac{Sd}{M}\times 100\%$$ (3)

where Sd is the standard deviation of the *Q*_10_ values, M is the mean of the *Q*_10_ values.

*Q*_10_ values were estimated for short-term (seasonal *Q*_10_, *Q*_10s_) and long-term (annual *Q*_10_, *Q*_10a_) by using monthly and annual (the whole growing season in our study) data sets of Rs for each community site, respectively [11]. We calculated *Q*_10s_ based on the daily Rs data for each month during the 2011–2012 growing seasons and calculated *Q*_10a_ based on the Rs data of the whole growing season for a single year and for both years.

A two-way ANOVA at *α*<0.05 was used to test for significant differences between *Q*_10s_ and *Q*_10a_ of the four vegetation types within a year and over the two years. The Duncan’s multiple range test was used to test for significant differences in *Q*_10_ values within each year, between the two years and among the four vegetation types. Linear regression analysis was used to assess relationships between *Q*_10s_ values and mean Ts and Ws at each site. The combined effects of Ts and Ws on *Q*_10_ were explored with multivariate non-linear regression analysis. All statistics were conducted by using SPSS version 16.0.

## Results

### Temporal variation of *Q*_10_

**Fig 3:**
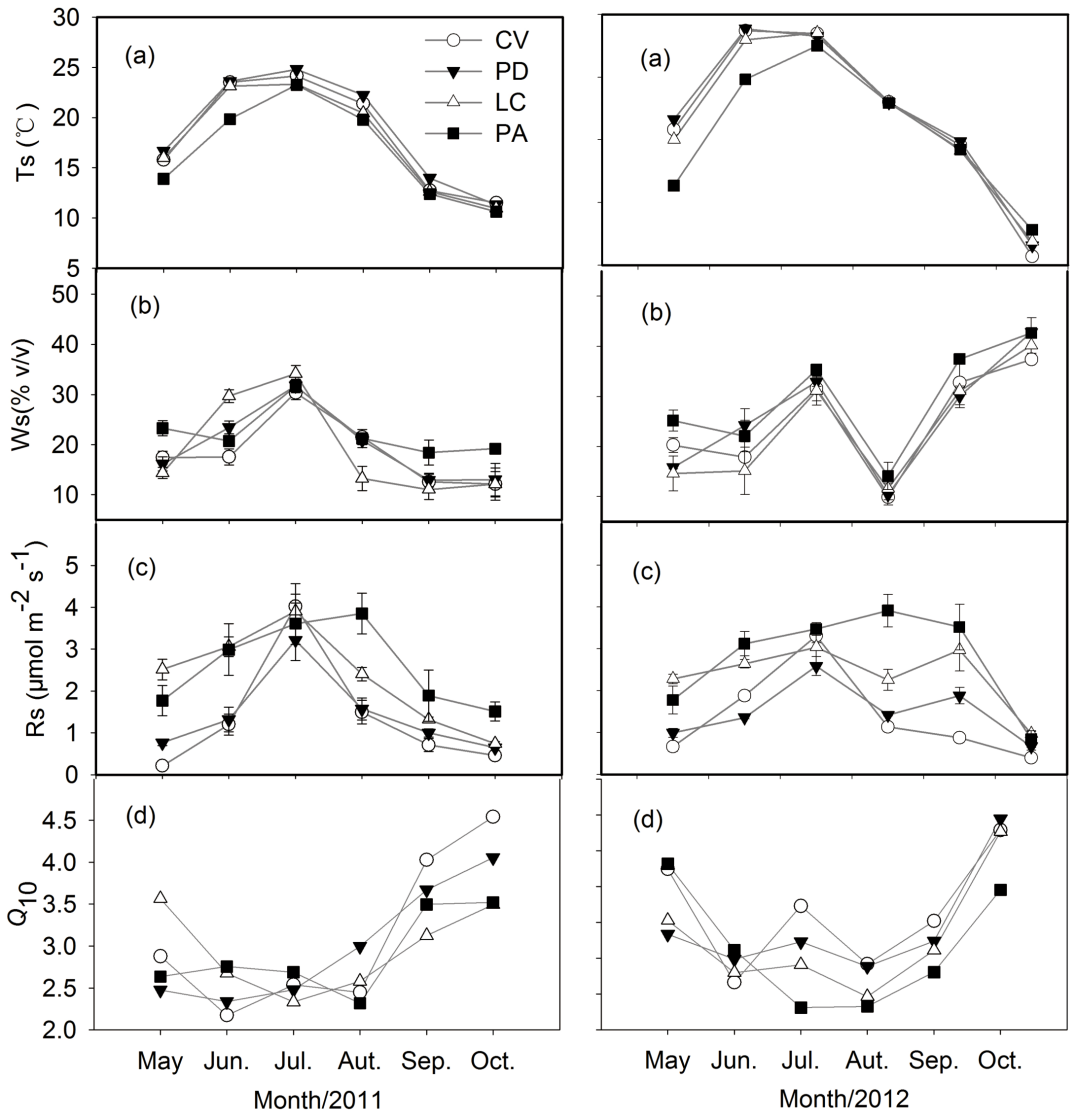
Seasonal variations of soil temperature (Ts), soil water content (Ws), soil respiration rate and *Q*_10_ at four communities during the 2011–2012 growing seasons. Error bars represent standard errors of the mean. The site codes were shown in Table 1.

The *Q*_10s_ of all four different vegetation sites exhibited large variations with time of the growing season. The Q_10s_ values ranged from 2.16 to 4.54, 2.34 to 4.45, 1.97 to 4.23 and 1.81 to 3.82 in the CV, PD, LC and PA site, with the monthly coefficients of variation of 26.93%, 23.65%, 22.94% and 24.16%, respectively. However, the *Q*_10s_ values showed a similar pattern among four vegetation types with a minimum value (1.81–2.34) occurring during summer and a peak value (3.82–4.54) occurring either in spring or autumn. Generally, the seasonal pattern of *Q*_10_ at the four community sites showed an opposite trend to soil temperature, but no significant correlation with soil moisture (Fig 3).

**Fig 4:**
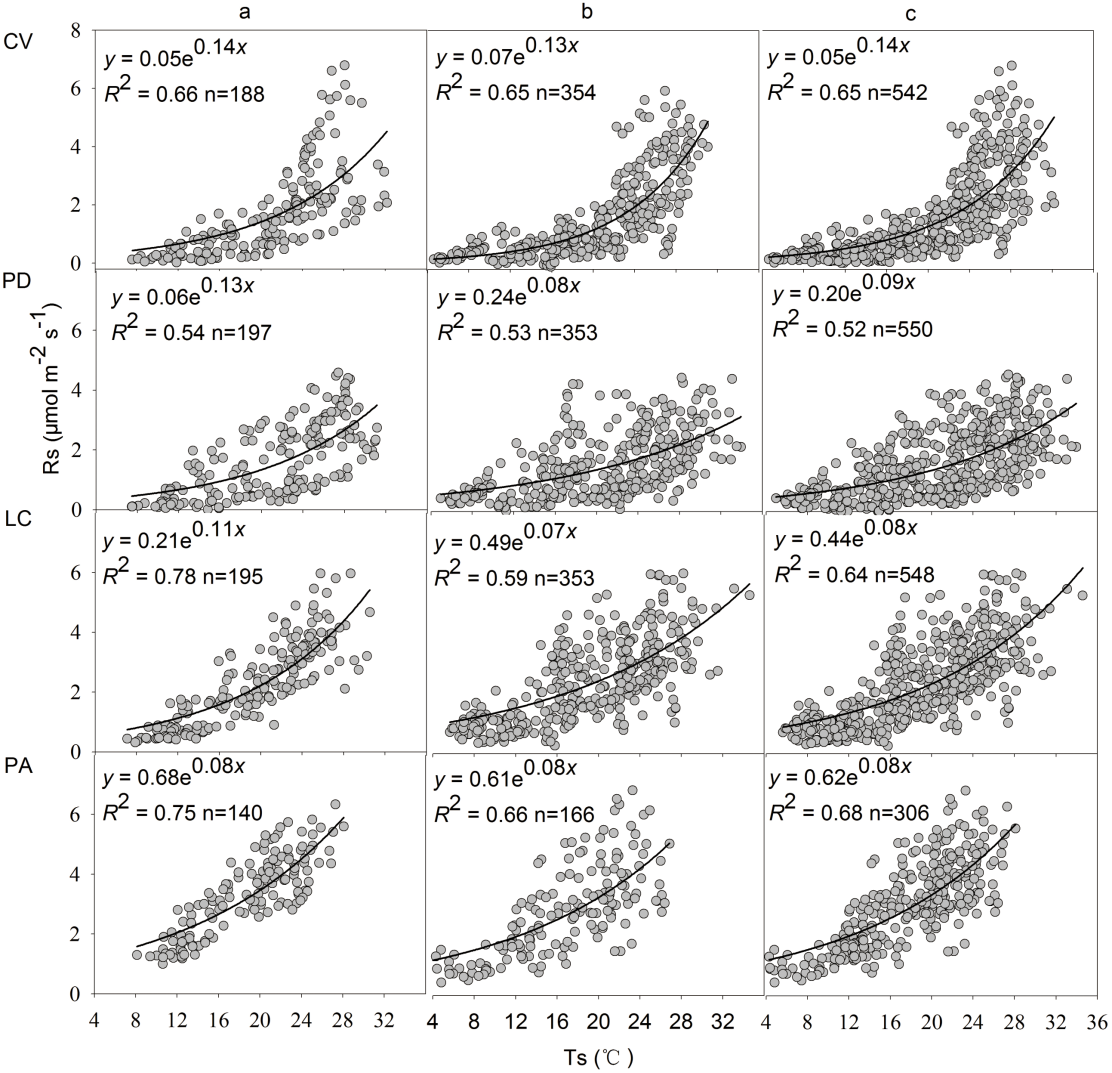
Relationships between soil respiration rate and soil temperature at 10 cm depth during the 2011–2012 growing seasons. (a) the year of 2011; (b) the year of 2012; (c) the years of 2011 and 2012. See Table 1 for the site codes.

Rs of the four vegetation types were strongly correlated with Ts within each growing season and over the two years combined (Fig 4). The *Q*_10a_ values in 2011 and 2012 and over the two years was 3.12–4.22, 2.23–3.67, 2.01–3.00 and 2.23 for CV, PD, LC and PA site, respectively (Table 2). The *Q*_10a_ values in 2011 (ranges: 2.23–4.22) were slightly higher than those in 2012 (ranges: 2.01–3.67) (Table 2), but there was no significant difference of *Q*_10a_ values across four vegetation types between the year 2011 and 2012.

**Table 2:** The means of seasonal *Q*_10_ values (*Q*_10s_) and the annual *Q*_10_ values (*Q*_10a_) during the growing seasons in each measured year for each community.

Sites	2011		2012		2011–2012	
	*Q*_10s_	*Q*_10a_	*Q*_10s_	*Q*_10a_	*Q*_10s_	*Q*_10a_
CV	3.10 ±0.39a	4.22 ±0.23a	3.14 ±0.33a	3.67 ±0.24a	3.12±0.24a	4.06 ±0.19a
PD	3.00 ±0.29a	3.67 ±0.24b	2.94 ±0.31a	2.23 ±0.25b	2.97±0.20a	2.46 ±0.33b
LC	2.96 ±0.21a	3.00 ±0.26b	2.76 ±0.33a	2.01 ±0.14b	2.86±0.19a	2.23 ±0.21b
PA	2.90 ±0.20a	2.23 ±0.18c	2.64 ±0.34a	2.23 ±0.16b	2.77±0.19a	2.23 ±0.13b

Values are the means ± SE. One-way analysis of variance (ANOVA) was used to compare the seasonal *Q*_10_ values and the annual *Q*_10_ values among the four vegetation types. Different letters indicate significant differences among the vegetation types (*p*< 0.05). See Table 1 for the site codes.

### Spatial variation of *Q*_10_

CV, PD, LC and PA are four most common vegetation communities within the same grassland ecosystem in the Songnen Plain, and the distribution of the four vegetation communities and belowground soil properties are markedly patchy in a regional scale (Table 1).

The mean *Q*_10s_ at the CV site (3.10 in 2011, 3.14 in 2012) was slight higher than the other three communities (2.23–3.0) (Table 2). However, there was no significant difference of the *Q*_10s_ among the four vegetation types (Table 2). The *Q*_10a_ values varied among the four vegetation communities, ranging from 2.23 (Pa) to 4.22 (CV) in 2011 and from 2.01 (Lc) to 3.67 (CV) in 2012, with the spatial Cvs of 26.21% and 30.13%, respectively. The *Q*_10a_ at the CV site (3.67–4.22) was significantly higher than the other three communities in 2011 (2.23–3.67) and 2012 (2.01–2.23) and over the two years (2.23–2.46) (*p*<0.05) (Table 2).

### Effects of soil moisture and temperature on *Q*_10_

**Fig 5:**
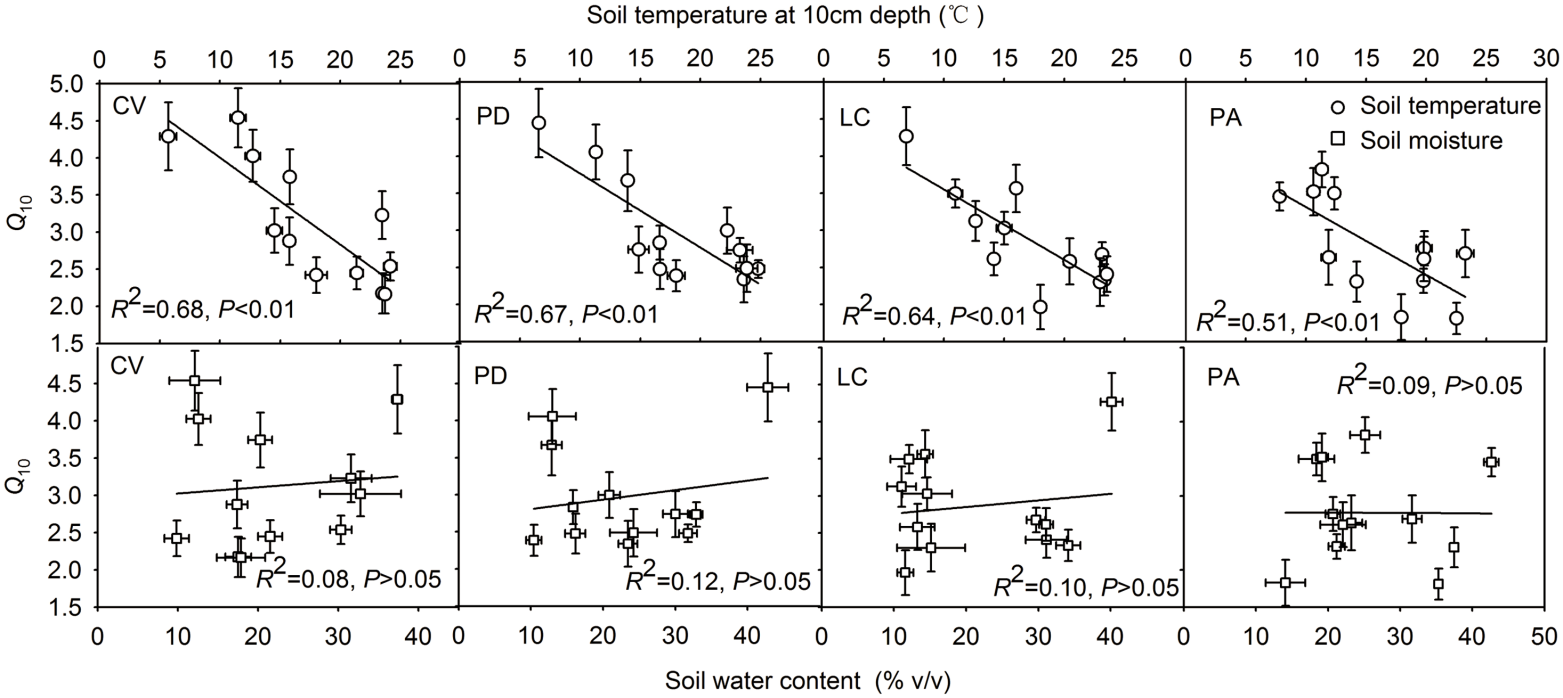
The relationship between *Q*_10_ and soil temperature and soil moisture at 10 cm depth, respectively. See Table 1 for the site codes.

**Fig 6:**
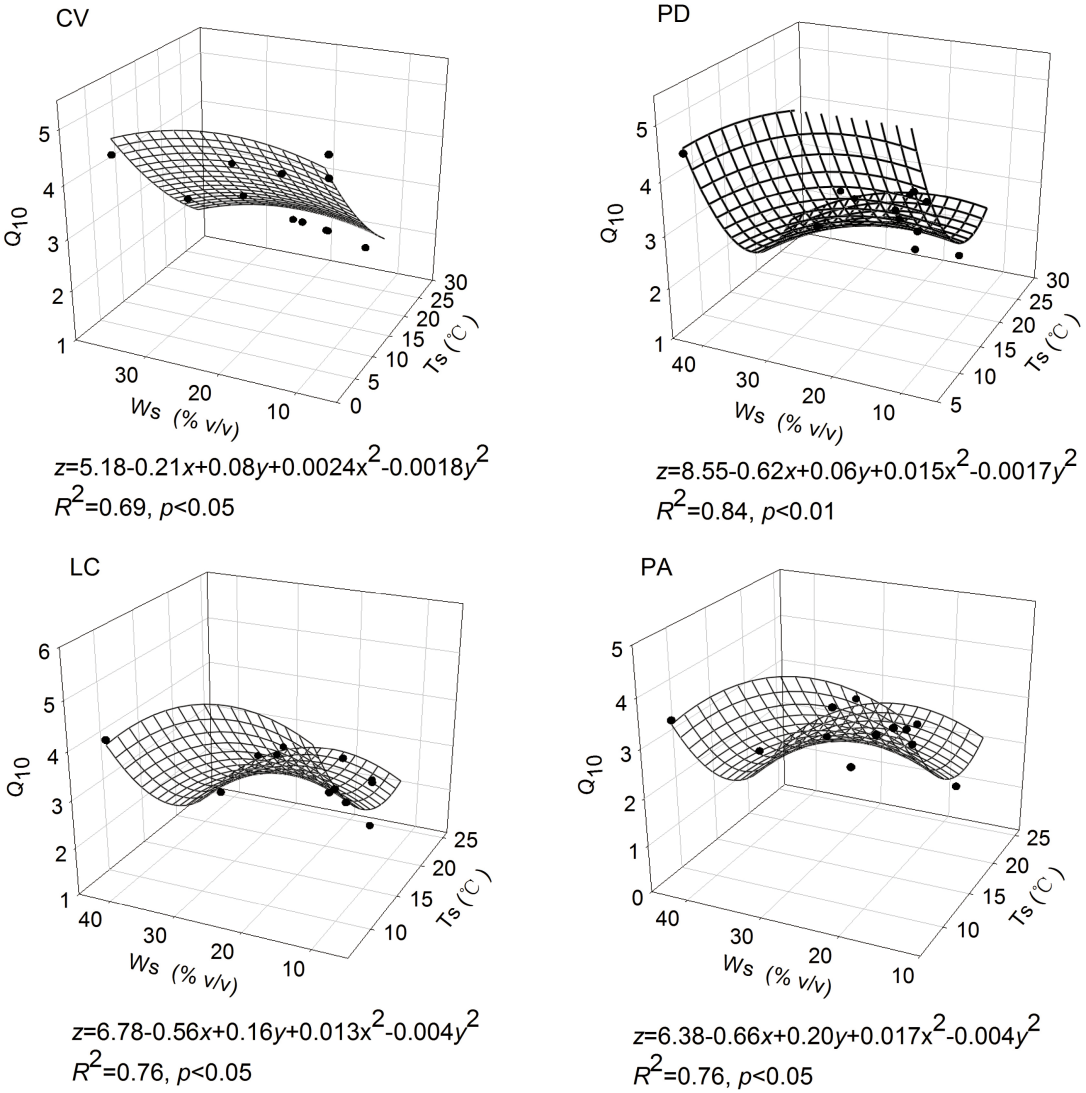
The interactive effect between *Q*_10_ and soil temperature and soil moisture at 10 cm depth. See Table 1 for the site codes.

The relationships between *Q*_10_ and soil temperature and soil moisture were modeled using linear regression, respectively (Fig 5). The *Q*_10_ value was significantly negatively corrected with soil temperature at all four vegetation sites (*p*<0.01). There were no significant effect of soil moisture on *Q*_10_ value during the two growing seasons at all sites (*P*>0.05). However, the soil temperature and moisture had interactive effects on the variations of *Q*_10_ value and explained 69%, 84%, 76% and 76% of the changes in the *Q*_10_ at all sites, respectively (Fig 6).

## Discussion

### Temporal variation of *Q*_10_

In the present study, the higher *Q*_10_ value in spring and autumn was contrary to that in summer with a lower *Q*_10_ (Fig 3), suggesting that Rs showed higher sensitivity to temperature during the colder months than during the warmer months. The seasonal trend of *Q*_10_ values in our study is similar to those reported for forest ecosystems [11, 24–26], grassland and cropland ecosystems [27, 28]. The major reason for the seasonal variation of *Q*_10_ is that, at the beginning of the growing season, the soil temperature in the surface layer is well coupled to the change of the air temperature and the diurnal variation is consequently high, so the uppermost part of soil profile is biologically active [29]. As a result, *Q*_10_ value is high in spring. As the season progresses, the soil temperature in the deeper soil profile increases, thus, deeper soil becomes biologically active. However, the magnitude of the diurnal variation in soil temperature become smaller in the deeper part of soil profile, so the sensitivity of soil respiration to diurnal variations in temperature decreases, which lead to a lower *Q*_10_. At the end of the growing season, a new litter layer of decomposing biomass on topsoil might provide a fresh energy source for microorganisms, which could enhance the soil microbial activities. Moreover, the variation of diurnal temperature on the surface soil in late autumn was as high as that in early spring, which makes the soil microorganisms more active [30], thus may cause the higher *Q*_10_ values as we observed in the autumn. In addition, studies suggested that the seasonal variation in *Q*_10_ probably resulted, in part, from the distinct seasonal patterns of physiological activity associated with root growth [31]. The higher *Q*_10_ in spring and autumn in this study may also be attributed to a surge in root respiration caused by the greater physiological activity of root growth [32]. At the starting of the growing season, along with soil temperature rising, the roots grew rapidly and absorbed water and nutrient to support the growth of above ground parts [33]; while during the autumn, as cessation of plant above-ground parts, accumulated roots reached the second peak to supply the growth of above-ground parts of the plant in next spring [34]. Therefore, at both the beginning and the end of the growing season, the rapid growth and large amount of roots may stimulate the response of Rs to temperature, thus lead to the higher *Q*_10_ values. Furthermore, Sampson *et al*. and Wang *et al*. suggested that seasonal variation in the *Q*_10_ of Rs among different ecosystems could be related to seasonal differences in photosynthesis [31, 35]. Another study found that variations in plant phenology could significantly contribute to the variations of the seasonal *Q*_10_ values [13], which were attributed to seasonal differences in plant photosynthesis.

### Spatial variation of *Q*_10_

Our results indicate that responses of soil CO_2_ emission among different plant communities within the same grassland ecosystem to the variations of soil temperature may vary as indicated by their different *Q*_10a_ values. One cause for the Rs variation could be the uniquely phenological patterns of the different plant communities with the process of plant succession, which may significantly influence the *Q*_10_ values [13]. In our study sites, there were substantially spatial variations in *Q*_10_ of Rs at the four vegetation communities in different successional stages. The pioneer CV community had the significant higher annual *Q*_10_ values than the other three communities, suggesting that the soil respiration of CV in the earlier successional stage was more sensitive to change in temperature. *Chloris virgata* is the only therophyte (annual plant) whereas the other three dominant plant species are perennial in the latter successional stage. A previous study found that seasonal vegetation activity could exert dominant controlling force on the *Q*_10_ across different biomes, which imply the ecological linkage between soil processes and plant physiological processes [29]. Phenological patterns of belowground root processes in therophyte and perennial vegetation are considerably different. Perennial species, with more extensive root systems and a long growth period, generally have better adaptability to changes in temperature. However, annual or seasonal therophytes, with short life cycles, always depend strongly upon the variation of temperature, as they need to sprout, flower, produce seed, and die, during the warmer months of the year. In the CV site, as seeds always sprout in the late June or July, the soil CO_2_ emission in the spring was paralleled to the decomposition of soil carbon matter. Soil warming, especially during the short summer, can enhance root growth sharply, which leads to the enhancement of plant-derived CO_2_ release from root respiration, and results in a significant increase in the soil CO_2_ emission [36]. As a result, soil respiration at the CV site may change from heterotrophic dominated activity to root driven activities. Such abrupt shifts may lead to a higher *Q*_10_ in the pioneer annual plant communities compared to the three perennials in advanced successional stages.

Another cause for the effect of vegetation type on *Q*_10_ values is possibly attributed to the different microbial communities and the amount of SOC among different vegetation communities with the process of plant succession [36], which would have a significant effect on the response of Rs to temperature. The labile SOC components provide important substrate for soil microbial respiration. Therefore, a change of the labile carbon in the topsoil could affect the soil microbial activities [37], causing the temperature sensitivity of SOC decomposition to vary among soils with different labile SOC contents. Our study indicates that perennial plants at advanced-succession stages have higher SOC content and lower *Q*_10_ values than the annual plants (Table 1). When the early and mid-succession plants are replaced by the late-succession plants, the SOC content and recalcitrant carbon reservoirs in later plants increase by accumulating more stable carbon into the soil [38]. The recalcitrant carbon is less sensitive to temperature than labile carbon [39,40], which could further explain the higher *Q*_10_ in annual plants than in perennial plants.

Overall, the *Q*_10_ values of the four communities show a tendency to decrease with the process of plant succession. Since the annual plants are more sensitive to the change of temperature, a small change in temperature may greatly affect the magnitude of soil CO_2_ efflux. The potential increase in CO_2_ release from the soil caused by future rising temperature may have a positive feedback effect on the atmospheric CO_2_ and global climate change [21]. Moreover, studies suggested that the ability of large perennials dedicated to bioenergy production to sequester substantial amounts of carbon [41,42]. Thus, the conversion of annual plants to perennial plants with the plant succession can be expected to increase carbon stored in above- and below-ground biomass and in soil organic matter because of their perennial nature and greater root biomass [42]. Therefore, fencing and vegetation protection to promote the plant progressive succession could be important to maximize C storage for this meadow steppe ecosystem.

### Effects of soil moisture and temperature on *Q*_10_

*Q*_10_ is generally affected greatly by soil temperature and moisture. A number of studies reported that there was a negative correlation between *Q*_10_ and temperature [43,44], as was observed in the present study. The studied meadow steppe sites, including four vegetation types, conferred a significant regression equation (*p*<0.05, Fig 5) to the relationship between *Q*_10_ and soil temperature. The decrease in *Q*_10_ value with increasing temperature might be related to the transition from adaption of enzymatic activity at low temperatures to limitation of substrate availability at high temperatures [45]. Lower temperature could cause the active microbes become dormant, decrease the species richness of microbes, and potentially lead to a higher *Q*_10_ value than expected [11]. Moreover, microbial populations could be affected more significantly at lower temperatures than at higher temperature, which also result in higher *Q*_10_ values with decreasing temperature [43]. In addition, the supply of substrate can also be a limiting factor at high temperatures, either through the effect of soil moisture and temperature on substrate availability or through the temperature sensitivity of the enzyme [46]. These interactions tended to produce a high *Q*_10_ value at the low temperature condition for biological activity.

Soil moisture is expected to influence the response of soil respiration to temperature [47], but the interpretations are complicated [17, 48]. Some studies showed that the soil water content and *Q*_10_ values might positively or negatively correlated with different water regimes [47–50], whereas others reported that soil moisture was not the limiting factors of the variations of *Q*_10_ [14, 27, 36]. However, the previous studies mainly used liner regression to explain the independent contributions of soil water content or soil temperature on the variations of *Q*_10_. In our study, there was no significant correlation between soil water content and seasonal *Q*_10_ values with liner regression (Fig 5). However, the multivariate nonlinear regression analysis with soil temperature and moisture in the equation could well explain the variations of *Q*_10_ at all sites (*R*^2^ = 0.69–0.84; Fig 6), which indicated that soil moisture might also be a potential contributor to the seasonal variations of *Q*_10_, and the soil temperature and soil moisture may have a confounding effects on *Q*_10_. Additionally, previous studies showed that the effects of Ts and Ws on *Q*_10_ were often interactive, especially in the field-based Rs measurement conditions [7,51,52]. Since *Q*_10_ was not only a reflection of temperature sensitivity, but also a combined response to fluctuations in temperature, soil moisture conditions, plant root biomass and activity, microbial populations and enzyme activity, as well as other unknown factors [30], the nonlinear regressions we built in the present study will help explain the relationship between soil respiration sensitivity to temperature and soil moisture, and could be used as model equations for the further study in this meadow steppe. Because soil moisture would likely be a potential factor to influence the seasonal variations of *Q*_10_ with climate change, soil moisture or precipitation should be treated as important controlling factors in further soil respiration measurements, especially for semi-arid or arid regions.

## Conclusions

Our study analyzed temporal and spatial variations in *Q*_10_ of Rs and their controlling factors in temperate meadow steppe of the Songnen Plain, China. On the spatial scale, *Q*_10_ value was significantly affected by vegetation type, with the annual plant community having higher *Q*_10_ values than the perennial plants, suggesting that fencing and vegetation protection to promote the plant progressive succession could be important to maximize C storage for this meadow steppe ecosystem under conditions of global climate change and global warming. *Q*_10_ values showed large variations seasonally, with a high *Q*_10_ value both in spring and autumn, but a low *Q*_10_ value in summer. The soil temperature and moisture had interactive effects on the temporal variations of *Q*_10_ values. The soil temperature was the dominant factor influencing *Q*_10_ values, while soil moisture was a potential contributor to the variations in *Q*_10_. Because of the significant temporal and spatial variations of *Q*_10_ in this meadow steppe ecosystem, further studies on the temporal and spatial pattern of *Q*_10_ and its controlling factors, as well as the function for revealing such patterns, are required in order to predict future responses of Rs to climate change and improve the precision of carbon budget estimations on regional scales.
